# Non-Invasive Luciferase Imaging of Type I Interferon Induction in a Transgenic Mouse Model of Biomaterial Associated Bacterial Infections: Microbial Specificity and Inter-Bacterial Species Interactions

**DOI:** 10.3390/microorganisms8101624

**Published:** 2020-10-21

**Authors:** Muhammad Imran Rahim, Andreas Winkel, Stefan Lienenklaus, Nico S. Stumpp, Szymon P. Szafrański, Nadine Kommerein, Elmar Willbold, Janin Reifenrath, Peter P. Mueller, Michael Eisenburger, Meike Stiesch

**Affiliations:** 1 Department of Prosthetic Dentistry and Biomedical Materials Science, Hannover Medical School, Carl-Neuberg-Strasse 1, 30625 Hannover, Germany; Winkel.Andreas@mh-hannover.de (A.W.); stumpp.nico@mh-hannover.de (N.S.S.); Szafranski.Szymon@mh-hannover.de (S.P.S.); Kommerein.Nadine@mh-hannover.de (N.K.); Eisenburger.Michael@mh-hannover.de (M.E.); Stiesch.Meike@mh-hannover.de (M.S.); 2 Lower Saxony Centre for Biomedical Engineering, Implant Research and Development (NIFE), Stadtfelddamm 34, 30625 Hannover, Germany; Willbold.Elmar@MH-Hannover.de (E.W.); Reifenrath.Janin@mh-hannover.de (J.R.); 3 Institute of Laboratory Animal Science, Hannover Medical School, Carl-Neuberg-Strasse 1, 30625 Hannover, Germany; Lienenklaus.Stefan@mh-hannover.de; 4 Department of Orthopedic Surgery, Hannover Medical School, Carl-Neuberg-Strasse 1, 30625 Hannover, Germany; 5 Department of Chemical Biology, Helmholtz Center for Infection Research, Inhoffenstrasse 7, 38124 Braunschweig, Germany; Peter-Paul.Mueller@helmholtz-hzi.de

**Keywords:** animal model, biomaterial-associated infections, immune response, type I interferon, periodontal pathogens, bioluminescence, non-invasive in vivo imaging

## Abstract

The performance of biomaterials is often compromised by bacterial infections and subsequent inflammation. So far, the conventional analysis of inflammatory processes in vivo involves time-consuming histology and biochemical assays. The present study employed a mouse model where interferon beta (IFN-β) is monitored as a marker for non-invasive rapid detection of inflammation in implant-related infections. The mouse model comprises subcutaneous implantation of morphologically modified titanium, followed by experimental infections with four taxonomically diverse oral bacteria: *Streptococcus oralis, Aggregatibacter actinomycetemcomitans, Porphyromonas gingivalis* and *Treponema denticola* (as mono culture or selected mixed-culture). IFN-β expression increased upon infections depending on the type of pathogen and was prolonged by the presence of the implant. IFN-β expression kinetics reduced with two mixed species infections when compared with the single species. Histological and confocal microscopy confirmed pathogen-specific infiltration of inflammatory cells at the implant-tissue interface. This was observed mainly in the vicinity of infected implants and was, in contrast to interferon expression, higher in infections with dual species. In summary, this non-invasive mouse model can be used to quantify longitudinally host inflammation in real time and suggests that the polymicrobial character of infection, highly relevant to clinical situations, has complex effects on host immunity.

## 1. Introduction

Medical implants can restore functions of diseased or damaged organs [1,2]. Despite medical advantages of implants, infectious bacterial biofilms may colonize biomaterials and lead to serious complications [3]. Such biofilms develop significant resistance to antibiotic treatment, followed by aggressive inflammation, and tissue destruction [4]. In contrast to biomaterials implanted elsewhere in the body, dental implants are constantly exposed to the oral microbiome, comprising about 700 different bacterial species, viruses, archaea, fungi and protozoans [5,6,7]. Most species of this oral microbiome are commensal microflora which are not problematic for the host since they maintain a symbiotic homeostatic balance with the host immune system [8,9]. However, a dysbiosis (microbial imbalance) can develop because of a systemic condition, poor oral health, hygiene, compromised immunity or smoking. In such case opportunistic pathogens (usually Gram-negative and occasionally Gram-positive) increase in numbers, whole biofilm expand and become more pathogenic [10]. The homeostatic balance is disrupted since immunity can no longer control biofilm and an intense destructive inflammatory reaction develops [11,12,13]. *Streptococcus oralis,* a commensal, is an early colonizer that supports subsequent colonization of disease-associated anaerobes [14,15]. *Aggregatibacter actinomycetemcomitans, Porphyromonas gingivalis* and *Treponema denticola* are the most frequently isolated bacterial species in dental implant infections and are associated with the destruction of host tissue [16]. These bacterial species form complex biofilms and trigger severe inflammatory reactions by acting synergistically or in cooperation [17]. Inflammatory reactions upon bacterial infections progress episodically, with active or inactive phases of tissue destruction [18,19]. This reflects diverse patterns of host-pathogen interactions. For further characterization, it is essential to extend investigations within living animals which has been restricted—mainly due to the lack of small animal models. The main difficulty in monitoring host immunity against biomaterials-related bacterial infections is that the majority of the animal models involve tissue extractions at selected time points, followed by time consuming immunological staining [20]. Therefore, non-invasive in vivo imaging of bioluminescent markers was employed to monitor inflammation within the same animal at multiple time points, thereby achieving more reproducible results while dramatically reducing the consumption of experimental animals [21,22]. Moreover, bioluminescent imaging significantly reduced the issues related to animal-to-animal variations and improved the biostatistical quality of data [23]. The non-invasive imaging of luciferase reporter gene expression in living animals after the immediate administration of luciferin with cooled charge-coupled device (CCCD) camera is a rapid and highly sensitive method [24]. The low luminescence background of healthy tissue, the fast activity of luciferase enzyme, and non-immunogenic characteristics of luciferin increase the utility of this method for in vivo imaging of temporal gene expression [25]. Exposure to bacterial pathogens elicits overt inflammation involving tightly controlled activation and/or release of an array of effectors comprising innate and adaptive immune cells which secrete cytokines that recruit, activate, and regulate inflammatory infiltrates [26]. Type I interferons (IFNs) are key determinants of host innate immune responses secreted by a broad range of cell types, including fibroblasts, leukocytes, and endothelial cells [27]. Previous studies in human patients and small animals have shown that type I IFNs are expressed in infections by different periodontal pathogens, however, little is known about the activation of interferon in bacterial infections associated with biomaterials [28,29]. Previously, a luciferase reporter mouse model which allows tracking of interferon beta (IFN-β) gene induction in vivo was established. Recently, this mouse model was used successfully for the non-invasive imaging of IFN-β upon infections with *Pseudomonas aeruginosa* biofilms on degradable implants [22]. This study suggested that IFN-β luciferase reporter mouse model could be used as a highly sensitive reporter system to investigate host inflammation against biomaterial-related infections with diverse periodontal pathogens. The oral pathobionts we chose were *S. oralis*, *A. actinomycetemcomitans*, *P. gingivalis* and *T. denticola*. *A. actinomycetemcomitans* (DSM 11123) are associated with aggressive periodontitis [30]. *P. gingivalis* (DSM 20709) and *T. denticola* (DSM 14222) are considered major etiological agents to chronic periodontitis and were used in animal models [31,32,33]. To date, most of the previous studies were focused on single species biofilms, however, biofilms on teeth and dental implants are composed mostly from a variety of aerobic or anaerobic bacteria [7,34]. It indicates that cooperative or competitive interspecies interactions can have diverse influences on the response of the host tissue environment. Peri-implant inflammation is a tedious process which involves a malfunction interaction of implant, bacteria and host tissue which is hard to investigate in vivo. By using non-invasive imaging of IFN-β expression, an improved (advantageous) subcutaneous implant strategy and comparing it with the histological status of the tissue in a transgenic animal model, the effects of diverse oral pathogens on the host tissue and the differences which might occur with mixed species infections were investigated in this study.

## 2. Materials and Methods

### 2.1. Preparation of Implants

**Figure 1:**
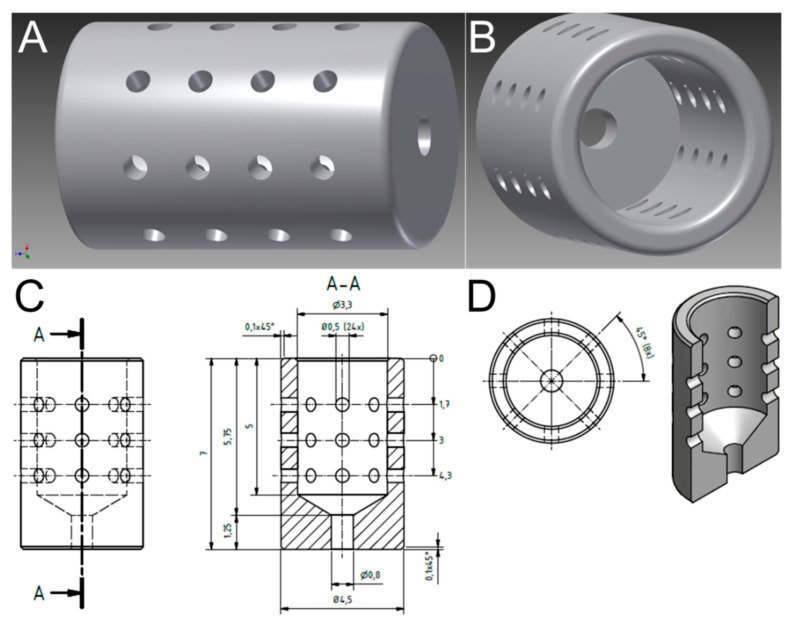
Details of cylindrical titanium implants. Holes were drilled in titanium cylinders (**A**,**B**). Cylindrical implant contains 24 holes, in the periphery each 0.5 mm in diameter, 1 hole at the bottom 0.8 mm and an opening at the top 3.3 mm (**C**,**D**).

For implantation, clinically established titanium (grade 4) was selected, as this is immunotolerant, biocompatible and is used in a variety of biomedical applications, including dentistry [35]. Titanium cylinders of 4.5 mm in diameter were purchased from L. Klein SA, Biel, Switzerland. At the Central Research Devices Service Unit, Hannover Medical School, Germany, rods of 7 mm in length were produced and further modified for in vivo applications into cups with an internal diameter of 3.3 mm. Beside a hole in the bottom of the cup (0.8 mm in diameter), 24 pores (0.5 mm each) were drilled into the periphery of the implants (see also Figure 1A–D).

### 2.2. Culture of Bacteria

*Streptococcus oralis* (ATCC 9811; American Type Culture Collection, Manassas, VA, USA) was cultured overnight on Tryptone Soy Agar (TSA) plates at 37 °C. A few colonies were inoculated into Tryptone Soy Broth (TSB) (Oxoid Limited, Hamsphire, UK) supplemented with 10% yeast extract (Carl Roth GmbH + Co. KG, Karlsruhe, Germany) and 50 mM glucose (Carl Roth GmbH + Co. KG, Karlsruhe, Germany) at 37 °C under constant shaking. *Aggregatibacter actinomycetemcomitans* (DSM 11123; German Collection of Microorganisms and Cell Cultures, Braunschweig, Germany) or *Porphyromonas gingivalis* (DSM 20709; German Collection of Microorganisms and Cell Cultures, Braunschweig, Germany) were cultured for 48 h on fastidious anaerobe agar (FAA) plates (LabM, Heywood, UK), supplemented with 5% sheep blood at 37 °C under anaerobic conditions (80% N_2_, 10% H_2_, 10% CO_2_). A few colonies of *A. actinomycetemcomitans* or *P. gingivalis* were inoculated overnight into brain heart infusion medium (BHI; Oxoid, Wesl, Germany) supplemented with 10 μg/mL vitamin K (Roth, Karlsruhe, Germany) under anaerobic conditions [36]. *Treponema denticola* (DSM 14222; German Collection of Microorganisms and Cell Cultures, Braunschweig, Germany) was cultured in new oral spirochete (NOS) medium at 37 °C for 72 h under static anaerobic conditions [37].

### 2.3. In Vivo Implantation and Infection Procedures

Animal experiments were performed with permission number: 33.12-42502-04-17/2580 from the Lower Saxony State Office for Consumer Protection and Food Safety, Germany. A total of 39 female 8–12 weeks old IFN-β reporter mice (C.Balb/c1-Ifnb1tm1.2Lien, bred at the Central Animal Facility, Hannover Medical School, Germany) weighing 22 ± 2 g were implemented in the study. Animals were divided into 13 different groups (Supplementary Table S1). Three animals were used for each group; control group with implantations, groups only with surgical pouches and infections, groups with titanium implants and infections with *S. oralis*, *A. actinomycetemcomitans*, *P. gingivalis* and *T. denticola* (alone or as selected combination of two species) (Supplementary Table S1). For surgical procedures, animals were anesthetized by intraperitoneal injection of 10 mg/kg ketamine (Anesketin; Albrecht GmBH, Aulendorf, Germany) and 4 mg/kg xylazine (Rompun; Bayer, Leverkusen, Germany). The implantation areas were shaved with a hair trimmer (Aesculap Suhl GmbH, Suhl, Germany) and disinfected with 70% ethanol. Three surgical pouches were created in the subcutaneous tissue with surgical scissors and tissue forceps (Fine Science Tools GmbH, Heidelberg, Germany). Titanium implants were gently inserted into these pouches and wounds were closed with a simple interrupted suture (Ethicon Vicryl, Johnson & Johnson Medical GmbH, Norderstedt, Germany). Within one hour after surgical procedure for implantation and wound closure, subcutaneous implants or surgical pouches without implants were infected with an injection of 5 μL of a bacterial suspension (as single or dual species with an infection dose of 1 × 10^8^ CFU/mL). For in vivo imaging, animals were anesthetized with an XGI-8 gas anesthesia unit (PerkinElmer, Waltham, MA, USA) using 2% isoflurane in oxygen mixture. D-luciferin (150 mg/kg) (PerkinElmer, Waltham, MA, USA) diluted in Dulbecco’s Phosphate Buffered Saline (PBS; Biochrom GmbH, Berlin, Germany) was intraperitoneally injected into the animals. Injections and surgery were all performed under a sterile hood. Bioluminescence imaging from animals was initiated at 15 min after luciferin injection under exposure conditions of 120 s with an in vivo imaging system (IVIS Spectrum CT; PerkinElmer, Waltham, MA, USA) on day 0, 2, 4, 6, 8, 11, 14 and 21. The bioluminescence around three implants per mouse was measured by manually drawing three different region of interest (ROI) with the Living Image Software (Version 4.5,PerkinElmer, Waltham, MA, USA) and the data were expressed as photon-flux (photons/s/cm^2^/sterdian) [38].

### 2.4. Histology and Biofilm Staining

After 21 days, explants were extracted from euthanized animals and stained using the LIVE/DEAD BacLight Bacterial Viability Kit (Life Technologies, Darmstadt, Germany). Explants were directly incubated for 30 min with SYTO^®^9 and propidium iodide (PI) (1:1000 dilutions in PBS) and fixed with 2.5% glutaraldehyde [36,39]. Various regions of stained explants were imaged with a confocal laser-scanning microscope (CLSM; Leica TCS SP2, Leica Microsystems, Mannheim, Germany) using 488 nm laser and an emission range of 500–545 nm for SYTO^®^9 or 590–680 nm for PI. The images stacks were acquired with an area of 1200 × 1200 μm^2^. Acquired images were processed with IMARIS software (Version 8.4, Bitplane, Zurich, Switzerland).

Tissues were isolated and fixed in 3.5% commercial buffered formalin (Otto Fischar, Saarbrücken, Germany) for two days at room temperature, dehydrated and embedded in paraffin using an automated embedding system (Pathcentre Tissue Processor, Shandon, Dreieich, Germany). Then, 5 µm thin sections were cut with a Leica RM 2155 microtome, mounted on poly-L-lysine coated glass slides and dried for at least 24 h at 37 °C. Prior to staining, the tissues were deparaffinized in xylene (3 × 10 min) and rehydrated in a series of decreasing concentrations of alcohol. For hematoxylin and eosin staining, sections were first rinsed in distilled water for 30 min, then stained for 5 min with Mayer’s hematoxylin (Merck, Darmstadt, Germany), rinsed in tap water for 10 min, then stained with 1% eosin (Merck, Darmstadt, Germany) for 3 min, dehydrated in a graded concentration series of ethanol and mounted in Eukitt (Labonord, Mönchengladbach, Germany) according to the standard procedure [40]. Pictures were taken with a Zeiss Axioscope 40 microscope combined with a Zeiss AxioCam Mrc digital Camera and Zeiss AxioVision software (Version 4.8, Zeiss, Oberkochen, Germany).

### 2.5. Statistical Analysis

SPSS Statistics (Version 26, IBM, Ehningen, Germany) was used for statistical analysis. The Kruskal–Wallis H test, a rank-based nonparametric test, with Dunn post hoc method was applied to test the null hypothesis between various groups: (a) non-infected implant vs various infected implants, (b) infections without implant vs peri-implant infections, and (c) single- vs dual-species infections both peri-implant and non-peri-implant. Three animals, each carrying three implants were used in each group. Luminescence data were analyzed implant-wise (*n* = 9) and pseudoreplication may have some minor effect. Symbols ***, *, and # indicated *p* < 0.001, *p* < 0.05, and significant *p* value prior to Bonferroni correction, respectively.

## 3. Results

### 3.1. Optimized Morphological Modifications Dedicated in Implant Design to Promote Prolonged and Reproducible Implant-Associated Infections

It is challenging to achieve reproducible and prolonged bacterial infections on implants since most of the injected bacteria get quickly eliminated by the host immune system. Titanium implants were modified into cylindrical porous morphologies so that implants provide safe niches to bacteria against the invasion of host immune cells (Figure 1). It was found that a diameter of 4.5 mm and length of 7 mm was appropriate for subcutaneous implantation in mice. Additionally, 24 pores (0.5 mm each) were drilled into each implant to (I) allow the bacterial biofilms inside the cylinder to interact with the host immune cells and (II) to capture luminescence from within the cylinders.

### 3.2. Biomaterial-Associated Infections Induce IFN-β Expression

**Figure 2:**
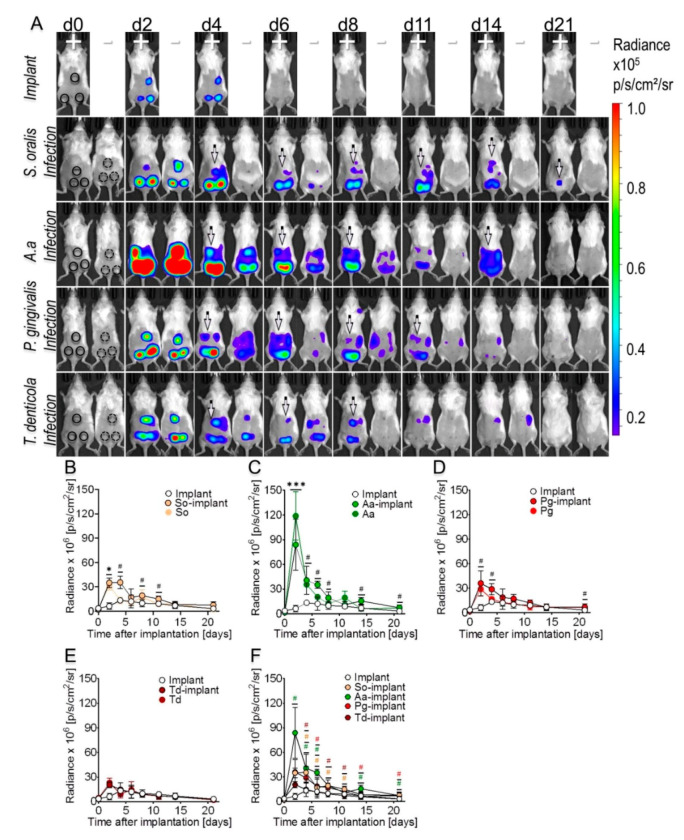
Interferon beta (IFN-β) induction during biomaterial-associated bacterial infections. (**A**) Black circles in each mouse show areas containing sterile implants or implants infected with *S. oralis* (*So*), *A. actinomycetemcomitans* (*Aa*), *P. gingivalis* (*Pg*) or *T. denticola* (*Td*) at day 0. Dashed black circles on day 0 show the regions for infected sham operated tissues. IFN luciferase luminescence intensity around implants (+) and sham-operated tissues (−) is indicated by bioluminescent patches (d2–d21). The illustration shows images from a typical single experiment selected from a group of three animals. In vivo measured bioluminescent signals from the sites of implantations were quantified and are shown here as radiance at the indicated time points (**B**–**F**). (**B**–**E**), illustrate IFN-β kinetics in response to *S. oralis* (yellow), *A. actinomycetemcomitans* (green), *P. gingivalis* (red), and *T. denticola* (dark red) infections of subcutaneous implants (circles with border), sham-operated tissues (circles without borders) and from sterile implants (white circles), respectively. (**F**) IFN-β luciferase activity from sterile implants and implants infected with different pathogens. Three animals, each carrying three implants were used in each group yielding *n* = 9 biological observations. Error bars indicate mean ± s.e.m. Significance values have been adjusted by the Bonferroni correction for multiple tests. Symbols ***, *, and # indicate *p* < 0.001, *p* < 0.05, and significant *p* values prior to Bonferroni correction, respectively. Black symbols represent significant *p* values between sterile and infected implants. Colored symbols indicate significant *p* values for each of the infected implants (*S. oralis* (yellow), *A. actinomycetemcomitans* (green), *P. gingivalis* (red) or *T. denticola* (dark red)) with sterile implant, respectively (**F**).

To determine a distinctive effect of various oral bacterial species on IFN-β induction, hollow titanium cylinders were implanted subcutaneously in highly sensitive luciferase reporter mice followed by infections with four taxonomically diverse pathogens, i.e., *Streptococcus oralis* from the Firmicutes phylum, proteobacterium *Aggregatibacter actinomycetemcomitans*, *Porphyromonas gingivalis* from the Bacteroidetes phylum or spirochaete *Treponema denticola* (Figure S1). Implantation and/or infection did not trigger immediate IFN-β expression (Figure 2A; d0). After two days, there was marginal luminescence, indicating IFN-β induction around sterile implants, and this was measurable until day 4 (Figure 2A; d2–d4). All bacterial infections induced high IFN-β reporter signals, both around titanium implants (+) and sham-operated tissues (−) (Figure 2A; d2). The IFN-β intensity was correlated with the type of pathogen (Figure 2A; d2; luminescent foci). *A. actinomycetemcomitans* infections caused the greatest IFN-β luciferase activity followed by *P. gingivalis, S. oralis* and *T. denticola* (Figure 2A; d2). From day 4 and onward, IFN-β luminescence around infected implants was higher than that of infected tissues without implants (Figure 2A; d4–d11, white arrows). Even as late as day 21, low IFN-β-related luminescence was detected around *S. oralis* infected implants (Figure 2A; d21, white arrow). The quantitative analysis of luminescence representing IFN-β induction confirmed the impact of the bacterial species and the presence of the implant in prolonging IFN-β expression (Figure 2B–F). IFN-β luciferase activity initially increased and then decreased gradually in the vicinity of sterile implants, infected implants and infected tissues without implants (Figure 2B–F). Compared to sterile implants, IFN-β luciferase luminescence intensity increased significantly upon infection in all cases. However, the luminescence from infected implants was greater and longer lasting than in infected tissues without implants (Figure 2A). Of the four tested bacterial species, *A. actinomycetemcomitans* was the most powerful and *T. denticola* the least powerful inducer of IFN-β (Figure 2F). Overall, IFN-β luciferase intensity and duration near infected implants increased in the presence of pathogens associated with acute infection.

### 3.3. Dual Species Bacterial Infections Decrease IFN-β Induction

**Figure 3:**
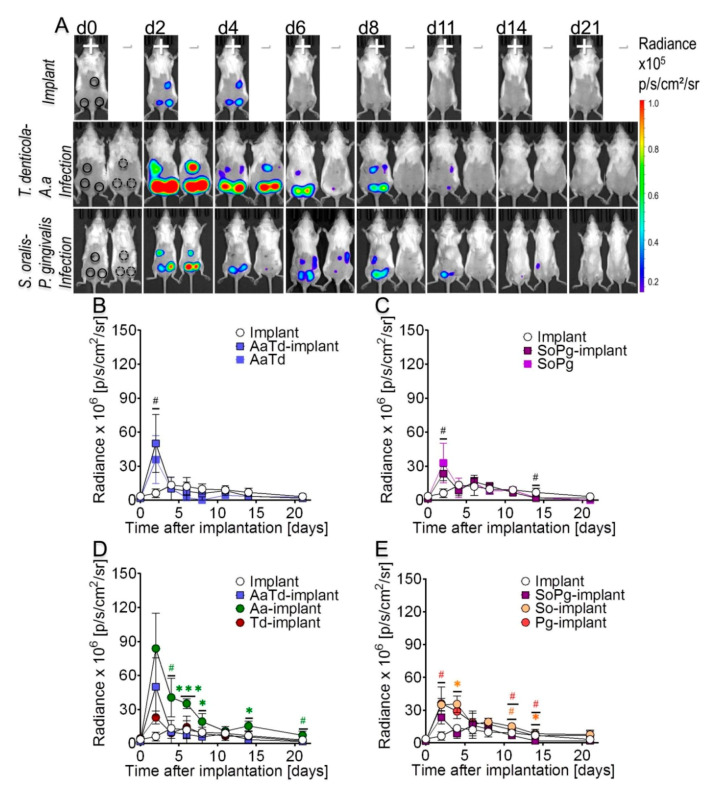
IFN-β induction upon dual species infections. (**A**) IFN-β luminescence measured at the indicated time points in days from sterile implants, infected implants (+), infected surgical pouches without implants (−). Bioluminescent images from a typical single experiment. Implantation sites were marked as region of interest and bioluminescent signals were quantified as radiance at the indicated time points (**B**,**C**). (**B**) IFN-β kinetics upon *A. actinomycetemcomitans-T. denticola* (AaTd) dual species infections of implants (blue squares with borders), tissues (blue squares). (**C**) IFN-β kinetics upon *S. oralis-P. gingivalis* (SoPg) dual species infections of implants (purple squares with borders), tissues (purple squares). (**D**) Comparison of Aa and Td implant infections as single species with AaTd dual species implant infections. (**E**) Comparison of So and Pg implant infections as single species with SoPg dual species infections. Three animals, each carrying three implants were used in each group yielding n = 9 biological observations. Error bars indicate mean ± s.e.m. Significance values have been adjusted by the Bonferroni correction for multiple tests. Symbols ***, *, and # indicate *p* < 0.001, *p* < 0.05, and significant *p* value prior to Bonferroni correction, respectively. Black symbols represent *p* values between sterile and infected implants (**B**,**C**). Colored symbols indicate *p* values between implants infected with single species to dual species infected implants (**D**,**E**).

To address more complex situations and to investigate synergistic interactions within two bacterial species, IFN-β expression was investigated after infections with dual species. Dual species infections were performed by mixing *S. oralis* (a commensal) or *A. actinomycetemcomitans* (associated to acute infections) with *P. gingivalis* or *T. denticola* (both commonly found in chronic infections). IFN-β expression around sterile implants was visible until day 4 (Figure 3A; d2–d4). IFN-β intensity from infected implants and infected sham-operated tissues was stronger than with sterile implants (Figure 3A; d2). *A. actinomycetemcomitans-T. denticola* (AaTd) induced high IFN-β expression compared to *S. oralis-P. gingivalis* (SoPg) dual species infections (Figure 3A; d2). Between days 4 and 11 the strongest intensities of IFN-β signaling in mice with or without implants were observed, particularly in the case of SoPg infected implants (Figure 3A; d4). Furthermore, IFN-β expression around infected implants gradually started to decrease and disappeared completely by day 11 (Figure 3A; d11). Quantitative analysis revealed higher interferon luminescence from infected implants compared to sterile implants and infected tissues (Figure 3B,C). Differential interferon expression indicated that *A. actinomycetemcomitans* (Aa) alone caused the greatest and *T. denticola* the lowest IFN-β expression, while *A. actinomycetemcomitans-T. denticola* (Aa-Td) infections had an intermediate effect, thereby indicating their mutual interaction in dual species infections (Figure 3D). IFN-β expression around implants infected with *S. oralis* or *P. gingivalis* as single species were partially significantly higher than those from combined dual species *S. oralis-P. gingivalis* infections (Figure 3E). Animals were regularly weighed and severe or life threatening deterioration of the overall health status was not observed at any time or with any treatment (Figure S2).

### 3.4. Cellular Responses during Implant-Related Infections

**Figure 4:**
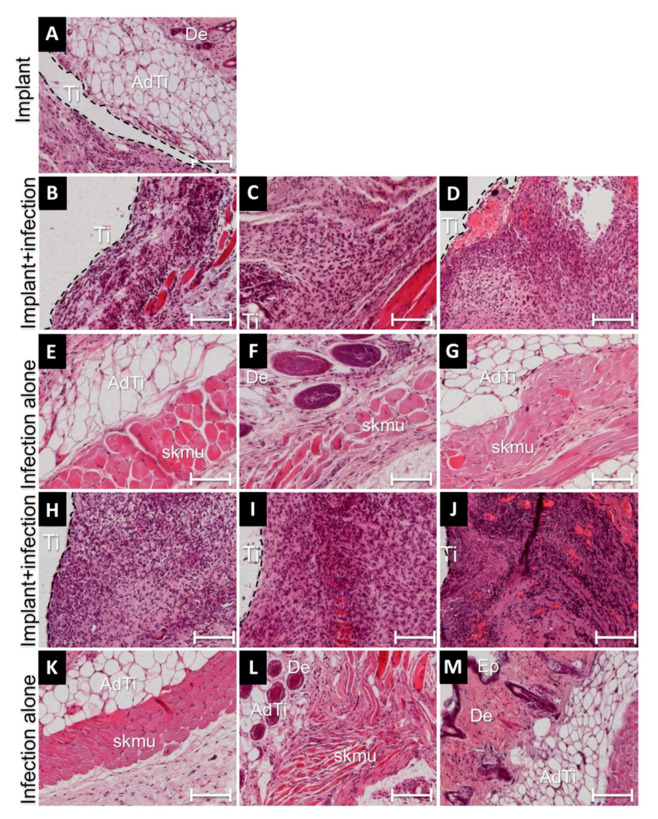
Histological evaluation around sterile and infected implants as well as in areas of infections without implants on day 21. Few immune cells are present at the sterile implant (Ti) tissue interface (**A**). Large numbers of inflammatory cells (blue nuclei) were present at tissues-implant (Ti) interfaces infected with *S. oralis* (**B**), *A. actinomycetemcomitans* (**C**), *P. gingivalis* (**D**) or *T. denticola* (**H**). Tissue infections without implants showed no signs of inflammation (**E**,**F**,**G**,**K**). Tissue-implant interfaces infected with dual species *S. oralis-P. gingivalis* (**I**) and *A. actinomycetemcomitans-T.denticola* (**J**) showed huge recruitment of inflammatory cells (blue nuclei). Dual species infected tissues without implants did not exhibit high recruitment of inflammatory cells (**L**,**M**). Abbreviations: Ti, site of implantation; AdTi, adipose tissue; skmu, skeletal muscles in the tissue, Ep, epidermis, De, dermis. The dashed border line indicate tissue-implant interface. The scale bar represents 100 micrometers in each case.

To investigate the recruitment of immune cells at sites of implantation and infection, hematoxylin and eosin staining of tissue thin sections was performed on day 21. The tissues near sterile implants exhibited mild recruitment of inflammatory cells (Figure 4A and Figure S3). The infiltration of inflammatory cells—predominantly foreign body giant cells (FBGCs)—and the proteinaceous network containing inflammatory cells indicative for fibrosis had increased in the infected peri-implant tissues compared to infected tissue without implants (Figure 4B–D,H and Figure S3). Only mild signs of inflammation were observed in the tissues with single species infections in the absence of implants (Figure 4E–G,K and Figure S3). Among single species infections, peri-implant tissues adjacent to *T. denticola* exhibited the highest infiltration of inflammatory cells, followed by *P. gingivalis*, *S. oralis* and *A. actinomycetemcomitans* (Figure 4H compared to Figure 4B–D and Figure S3). It is striking that the infiltration of inflammatory cells in the peri-implant tissues infected with any of the dual species combination was higher than in all single species infections (Figure 4I,J compared to Figure 4B–D,H and Figure S3). Tissues without implants infected with dual species did not show recruitment of immune cells (Figure 4L,M and Figure S3).

### 3.5. Tissue Implant Interfaces of Infected Implants Were Indicative of High Recruitment of Immune Cells

**Figure 5:**
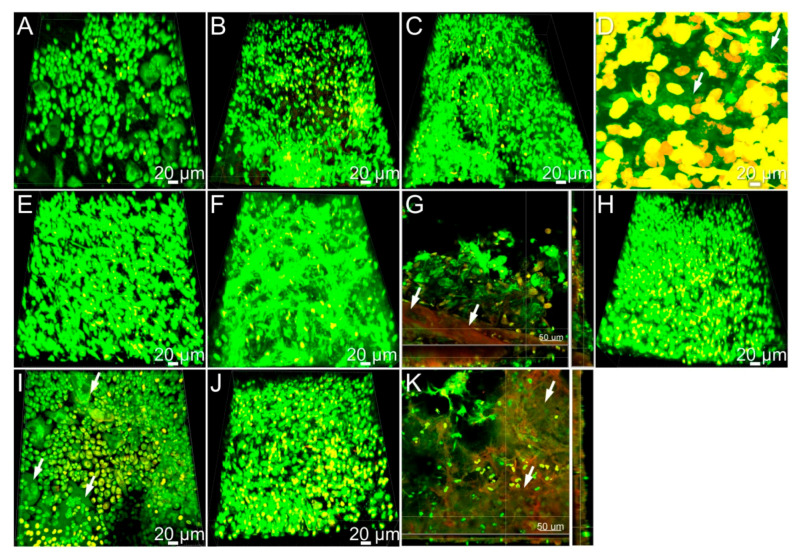
Bacterial infections promote accumulation of inflammatory cells in the implant-tissue interfaces. Confocal microscopic images of sterile titanium implants 21 days after implantation (**A**), *S. oralis* infected titanium implant surfaces (**B**), *A. actinomycetemcomitans* infected titanium implant surfaces (**C**), *A. actinomycetemcomitans* biofilms in the infected peri-implant tissues (**D**, white arrow), *P. gingivalis* infected titanium implant surfaces (**E**), *T. denticola* infected implant surfaces (**F**), fine fibrillary material interposed between the implant surface and the connective tissue (**G**, white arrows). *S. oralis-P. gingivalis* infected implants (**H**,**I**), *A. actinomycetemcomitans-T. denticola* infected implant surfaces (**J**). Thick layer of connective tissue (**K**, white arrow).

To obtain 3D view of the architecture of the tissue-implant interface, confocal laser scanning microscopy (CLSM) of explants at day 21 was performed. Tissue-implant interface near sterile titanium displayed a mild accumulation of host cells characteristic for a foreign body reaction (Figure 5A). The interfaces near infected implants were covered by host inflammatory cells (Figure 5B–K). *S. oralis* infected implant-tissue interfaces indicated a large number of host cells; however, bacterial biofilms were not visible (Figure 5B, green fluorescence). Tissue-implant interfaces near *A. actinomycetemcomitans* infected implants showed a high accumulation of host cells (Figure 5C) as well as bacterial biofilms (Figure 5D, white arrows). *P. gingivalis* infection caused high recruitment of host cells yet without visible traces of bacterial biofilms (Figure 5E). Tissue-implant interfaces in *T. denticola* infected implants indicated high accumulation of host cells (Figure 5F). The connective tissue-implant interface was characterized by a fine fibrillary material interposed between the implant surface and the connective tissue (Figure 5G, white arrows). Dual species implant infections comprising *S. oralis* and *P. gingivalis* caused high recruitment of immune cells predominantly foreign body giant cells (FBGCs) (Figure 5H,I, white arrows). *T. denticola* and *A. actinomycetemcomitans* infections showed typical inflammatory situation with a thick layer of connective tissue interposed between the implant surface and adjacent tissue (Figure 5J,K, white arrows). Overall, confocal microscopy evidenced characteristic signs of inflammation at tissue-implant interfaces and in some cases surviving bacteria.

## 4. Discussion

Direct non-invasive imaging of inflammation within living animals could increase the understanding of underlying processes by which the immune response is activated and sustained during implant-related infections. The involvement of inflammatory cytokines such as interferon upon biomaterial-related infections remains rather unexplored since most of the previous studies were focused more on the activation and recruitment of immune cells [41,42]. A critical factor in biofilm-related animal models remains the host immune system which in mice can easily kill the injected bacteria. Titanium is especially suitable as a material for dental implants as it is highly inert, biocompatible and resistant to the colonization by microorganisms [43]. Therefore, the important challenge to investigate bacterial infections on titanium implants in animal models remains the establishment of persistent infections with a much lower bacterial inoculum. To accept this challenge, either composition or design of implants could be modified to allow strong bacterial persistence even in the presence of an active host immune system [23]. Therefore, design of clinically applied titanium implants (grade 4) was modified into porous cylindrical shapes, implanted subcutaneously in wild types mice and then infected with luminescent *S. aureus* XEN 29 (data not shown) [44]. Overall, the implant design was such a success that *S. aureus* survived for a long time compared to no implant. One can now speculate that bacteria survive inside the cylindrical implants but are protected from excess immune cells attack. The pores seemed to essentially fabricate bacterial interaction with the surrounding tissue. The porous structures on the other hand allowed the monitoring of the tissue interferon response by imaging. These implants were implanted subcutaneously in triplicate per mouse to avoid high consumption of experimental animals and to reveal interferon induction arising purely from the interaction of injected bacteria. Subcutaneous tissue easily accommodated the insertion of large sized porous implants, persistent infections and the provision of large imaging areas. As an alternative, implantation and/or infection in the mice oral cavities could have been clinically more relevant. However, this procedure would have resulted in more stressful surgical procedures, imaging restrictions and mice oral microflora could have interfered with injected bacteria and disrupted the interpretation of final results. Although the composition of peri-implant bacterial infections had been extensively characterized, there is limited information available regarding the IFN-β response imaging of co-infections with mixture of diverse bacterial species. *P. gingivalis* colonizes the oral cavity by interacting with the oralis group of streptococci, so therefore one combination was composed by mixing *P. gingivalis* with *S. oralis* [45,46]. *A. actinomycetemcomitans* and *T. denticola* were reported to have the closest relationship in periodontal diseases, so that the second combination was composed of mixing them together [47]. The current study was able to non-invasively, quantitatively, and repetitively follow IFN-β expression on titanium implantation over an extended time period upon infections with diverse bacterial species on a real-time basis. This mouse model was advantageous in distinguishing IFN-β expression in the presence of implant alone, exclusive tissue infections or infections of implant materials. Moreover, IFN-β induction increased upon the invasion of pathogens or their products, so the inflammatory reactions from the surgical wounds were excluded in these analyses. The strength of IFN-β signal and the duration were highly dependent on the type of pathogen. Among the tested strains, *A. actinomycetemcomitans* infections emerged as the most powerful inducers of IFN-β thereby suggesting specificity in the host immune system towards particular bacterial species. Histology and confocal microscopy on day 21 indicated a large number of host immune cells in tissues near the infected implant. As reported in the literature, most of these cells seemed to be foreign body giant cells (FBGCs), which is a common indication for chronic inflammation upon biomaterial-associated infections [22,24,48,49]. Because of the infections with different bacteria or their selected combinations, we still found after 21 days a huge influx of the immune cells in the local tissue in the presence of an implant material. The IFN-β signal was prolonged in these cases (compared to the infections without implants). So the high recruitment of inflammatory cells in the peri-implant tissues is suggestive for three specific hypotheses: (a) bacteria at lower numbers were still present inside implanted materials, (b) not the bacteria but their products were still persisting within the implant materials, (c) and/or the implant material had prevented the tissue regeneration process resulting in high amount of inflammatory cells. To explain these hypotheses, we performed confocal microscopy of the tissues infected with single or dual species but could not visualize bacteria except for *A. actinomycetemcomitans* thereby indicating that bacteria at 21 day were too low to be detected by the system utilized here. However, the recruitment of immune cells was much higher around implants infected with dual species compared to single species infections which could lead to the hypothesis that the bacteria injected as dual species work together to decrease interferon inductions even though they were not microscopically detectable. Most research has been done with only single species infections, perhaps in an attempt to avoid higher complexity in their systems. However, there were different responses to single- and mixed-species infections thereby proving that some sort of interactions within different species and the immune system occur. Therefore it can be concluded that using multi-species infections is important to get a more realistic model for human infections. Histological findings from infected peri-implant tissues were in agreement to several other studies reporting that localized tissue responses were stronger to co-infection with different periodontal pathogens [50,51]. In agreement with these findings, mixed species infections with *Fusobacterium nucleatum* and *Porphyromonas gingivalis* in murine abscess model resulted in the formation of significantly larger sized lesions compared with primary infection with *P. gingivalis* alone [52]. Infection in murine abscess model with mixed *P. gingivalis* and *F. nucleatum* synergistically elicited a significantly greater lesion size and lethality compared with *P. gingivalis* alone [53]. Co-infection of mouse periodontal tissues with dual species *P. gingivalis* and *F. nucleatum* exhibited significantly more bone loss compared with mono-infections [54]. A study on rats has shown that polymicrobial oral infection with *P. gingivalis*, *T. denticola*, *Tannerella forsythia* and *F. nucleatum* induced stronger alveolar bone loss compared to infection by either bacterium alone [55]. IFN-β expression was higher around infected implants compared to only tissue infections suggesting that the presence of material was playing an important role towards the retention of prolonged infection as intended. Our results seem to explain in parts why the progression of inflammatory diseases at implants is more aggressive than that at natural teeth even though our findings do not provide direct proof to confirm these options. Overall, decreases in the type I interferon expression upon dual species infections leads to further advances in the treatment of complicated multispecies bacterial infections. The current study indicates diverse implications of dual species infections on host immunity, such as the reduction in interferon expression and high recruitment of immune cells compared to single species infections; however, future studies are critically required to investigate molecular mechanisms underlying such pathways in more detail.

## 5. Conclusions

The understanding of pathogenic mechanisms and the mutual influence of pathogens in cross-species bacterial communities within the host tissue environment is crucial for the diagnosis and development of novel therapeutic strategies. Bioluminescent imaging was used to analyze non-invasively IFN-β induction to biomaterial-associated infections by periodontal pathogens belonging to different groups (alone or as combination) in a mouse model. This mouse model allowed real time visualization of typically strong dynamics of IFN-β kinetics at multiple time points within the same animal. With this mouse model, we demonstrated the exquisite specificity of IFN-β kinetics to the bacteria belonging to different groups. We have found that—in contrast to single species—dual species infections decreased the expressions of IFN-β and increased the accumulation of inflammatory cells in the peri-implant tissues. This indicates that, despite the presence of high numbers of immune cells, dual species infections had different consequences to IFN induction at the local sites of implantation and infection. In this mouse model, it can already be seen that shifting from single species to complex dual species infections has distinct immunological consequences on interferon and localized tissue reactions.

## Supplementary Materials

The following are available online at https://www.mdpi.com/2076-2607/8/10/1624/s1, Figure S1: Selection of periodontal pathogens to investigate inflammatory responses against implant-related infections, Figure S2: Relative body weight of mice. Figure S3: Histological evaluation of skin around sterile implants, infected implants and infected tissue after 21 days. Table S1: Classification of animal groups employed for the current study.

## References

[1] Jinnouchi H., Torii S., Sakamoto A., Kolodgie F.D., Virmani R., Finn A.V. (2019). Fully bioresorbable vascular scaffolds: Lessons learned and future directions. Nat. Rev. Cardiol..

[2] Lee P.C., Dixon J. (2017). Medical devices for the treatment of obesity. Nat. Rev. Gastroenterol. Hepatol. Amp..

[3] Arciola C.R., Campoccia D., Montanaro L. (2018). Implant infections: Adhesion, biofilm formation and immune evasion. Nat. Rev. Microbiol..

[4] Piattelli A., Cosci F., Scarano A., Trisi P. (1995). Localized chronic suppurative bone infection as a sequel of peri-implantitis in a hydroxyapatite-coated dental implant. Biomaterials.

[5] Busscher H.J., Rinastiti M., Siswomihardjo W., Van Der Mei H.C. (2010). Biofilm Formation on Dental Restorative and Implant Materials. J. Dent. Res..

[6] Elter C., Heuer W., Demling A.P., Hannig M., Heidenblut T., Stiesch M. (2011). Comparative analysis of biofilm formation on dental implant abutments with respect to supra- and subgingival areas: Polytetrafluoroethylene versus titanium. Int. J. Prosthodont..

[7] Szafrański S.P., Kilian M., Yang I., Bei Der Wieden G., Winkel A., Hegermann J., Stiesch M. (2019). Diversity patterns of bacteriophages infecting Aggregatibacter and Haemophilus species across clades and niches. ISME J..

[8] Moutsopoulos N.M., Konkel J.E. (2018). Tissue-Specific Immunity at the Oral Mucosal Barrier. Trends Immunol..

[9] Lamont R.J., Koo H., Hajishengallis G. (2018). The oral microbiota: Dynamic communities and host interactions. Nat. Rev. Microbiol..

[10] Hajishengallis G., Lamont R.J. (2016). Dancing with the Stars: How Choreographed Bacterial Interactions Dictate Nososymbiocity and Give Rise to Keystone Pathogens, Accessory Pathogens, and Pathobionts. Trends Microbiol..

[11] Hajishengallis G. (2014). Periodontitis: From microbial immune subversion to systemic inflammation. Nat. Rev. Immunol..

[12] Bowen W.H., Burne R.A., Wu H., Koo H. (2018). Oral Biofilms: Pathogens, Matrix, and Polymicrobial Interactions in Microenvironments. Trends Microbiol..

[13] Olsen I., Lambris J.D., Hajishengallis G. (2017). Porphyromonas gingivalis disturbs host–commensal homeostasis by changing complement function. J. Oral Microbiol..

[14] Abranches J., Zeng L., Kajfasz J.K., Palmer S.R., Chakraborty B., Wen Z.T., Richards V.P., Brady L.J., Lemos J.A. (2018). Biology of Oral Streptococci. Microbiol. Spectr..

[15] Kolenbrander P.E., Palmer R.J., Periasamy S., Jakubovics N.S. (2010). Oral multispecies biofilm development and the key role of cell–cell distance. Nat. Rev. Microbiol..

[16] Pye A., Lockhart D., Dawson M., Murray C., Smith A. (2009). A review of dental implants and infection. J. Hosp. Infect..

[17] Giaouris E., Eheir E., Edesvaux M., Ehébraud M., Emøretrø T., Elangsrud S., Edoulgeraki A., Enychas G.-J., eKačániová M., Eczaczyk K. (2015). Intra- and inter-species interactions within biofilms of important foodborne bacterial pathogens. Front. Microbiol..

[18] Silva N., Abusleme L., Bravo D., Dutzan N., Garcia-Sesnich J., Vernal R., Hernández M., Gamonal J. (2015). Host response mechanisms in periodontal diseases. J. Appl. Oral Sci..

[19] Lamont R.J., Hajishengallis G. (2015). Polymicrobial synergy and dysbiosis in inflammatory disease. Trends Mol. Med..

[20] Mandakhalikar K.D., Rahmat J.N., Chiong E., Neoh K.G., Shen L., Tambyah P.A. (2018). Extraction and quantification of biofilm bacteria: Method optimized for urinary catheters. Sci. Rep..

[21] Mezzanotte L., Root M.V.T., Karatas H., Goun E.A., Löwik C.W. (2017). In Vivo Molecular Bioluminescence Imaging: New Tools and Applications. Trends Biotechnol..

[22] Rahim M.I., Babbar A., Lienenklaus S., Pils M.C., Rohde M. (2017). Degradable magnesium implant-associated infections by bacterial biofilms induce robust localized and systemic inflammatory reactions in a mouse model. Biomed. Mater..

[23] Rahim M.I., Rohde M., Rais B., Seitz J.-M., Mueller P.P. (2016). Susceptibility of metallic magnesium implants to bacterial biofilm infections. J. Biomed. Mater. Res. Part A.

[24] Rahim M.I., Szafrański S.P., Ingendoh-Tsakmakidis A., Stiesch M., Mueller P.P. (2020). Evidence for inoculum size and gas interfaces as critical factors in bacterial biofilm formation on magnesium implants in an animal model. Colloids Surf. B Biointerfaces.

[25] Hsieh C.-L., Xie Z., Liu Z.-Y., E Green J., Martin W.D., Datta M.W., Yeung F., Pan D., Chung L.W.K. (2005). A luciferase transgenic mouse model: Visualization of prostate development and its androgen responsiveness in live animals. J. Mol. Endocrinol..

[26] Sansonetti P.J., Di Santo J.P. (2007). Debugging how Bacteria Manipulate the Immune Response. Immunity.

[27] González-Navajas J.M., Lee J., David M., Raz E. (2012). Immunomodulatory functions of type I interferons. Nat. Rev. Immunol..

[28] Mizraji G., Nassar M., Segev H., Sharawi H., Eli-Berchoer L., Capucha T., Nir T., Tabib Y., Maimon A., Dishon S. (2017). Porphyromonas gingivalis Promotes Unrestrained Type I Interferon Production by Dysregulating TAM Signaling via MYD88 Degradation. Cell Rep..

[29] Wright H.J., Matthews J.B., Chapple I.L., Ling-Mountford N., Cooper P.R. (2008). Periodontitis associates with a type 1 IFN signature in peripheral blood neutrophils. J. Immunol..

[30] Henderson B., Ward J.M., Ready D. (2010). Aggregatibacter (*Actinobacillus*) actinomycetemcomitans: A triple A* periodontopathogen?. Periodontology 2000.

[31] Huang N., Gibson F.C. (2014). Immuno-Pathogenesis of Periodontal Disease: Current and Emerging Paradigms. Curr. Oral Health Rep..

[32] You M., Chan Y., Lacap-Bugler D., Huo Y.-B., Gao W., Leung W.K., Watt R.M. (2017). Oral Treponeme Major Surface Protein: Sequence Diversity and Distributions within Periodontal Niches. Mol. Oral Microbiol..

[33] Ehow K.Y., Esong K.P., Echan K.G. (2016). Porphyromonas gingivalis: An Overview of Periodontopathic Pathogen below the Gum Line. Front. Microbiol..

[34] Heuer W., Kettenring A., Stumpp S.N., Eberhard J., Gellermann E., Winkel A., Stiesch M. (2012). Metagenomic analysis of the peri-implant and periodontal microflora in patients with clinical signs of gingivitis or mucositis. Clin. Oral Investig..

[35] Williams D. (1981). Implants in dental and maxillofacial surgery. Biomaterials.

[36] Kommerein N., Stumpp S.N., Musken M., Ehlert N., Winkel A., Häussler S., Behrens P., Buettner F.F.R., Stiesch M. (2017). An oral multispecies biofilm model for high content screening applications. PLoS ONE.

[37] Chan E.C.S., Siboo R., Keng T., Psarra N., Hurley R., Cheng S.-L., Iugovaz I. (1993). Treponema denticola (ex Brumpt 1925) sp. nov., nom. rev., and Identification of New Spirochete Isolates from Periodontal Pockets. Int. J. Syst. Evol. Microbiol..

[38] Zheng J., Xu L., Zhou H., Zhang W., Chen Z. (2010). Quantitative analysis of cell tracing by in vivo imaging system. J. Huazhong Univ. Sci. Tech. Med. Sci..

[39] Kommerein N., Doll K., Stumpp N.S., Stiesch M. (2018). Development and characterization of an oral multispecies biofilm implant flow chamber model. PLoS ONE.

[40] Diekmann J., Bauer S., Weizbauer A., Willbold E., Windhagen H., Helmecke P., Lucas A., Reifenrath J., Nolte I., Ezechieli M. (2016). Examination of a biodegradable magnesium screw for the reconstruction of the anterior cruciate ligament: A pilot in vivo study in rabbits. Mater. Sci. Eng. C.

[41] Svensson S., Trobos M., Hoffman M., Norlindh B., Petronis S., Lausmaa J., Suska F., Thomsen P. (2015). A novel soft tissue model for biomaterial-associated infection and inflammation—Bacteriological, morphological and molecular observations. Biomaterials.

[42] Albrektsson T., Jemt T., Mölne J., Tengvall P., Wennerberg A. (2019). On inflammation-immunological balance theory-A critical apprehension of disease concepts around implants: Mucositis and marginal bone loss may represent normal conditions and not necessarily a state of disease. Clin. Implant. Dent. Relat. Res..

[43] Liu Y., Zhou Y., Jiang T., Liang Y.-D., Zhang Z., Wang Y.-N. (2017). Evaluation of the osseointegration of dental implants coated with calcium carbonate: An animal study. Int. J. Oral Sci..

[44] Croes M., Bakhshandeh S., Van Hengel I., Lietaert K., Van Kessel K., Pouran B., Van Der Wal B., Vogely H., Van Hecke W., Fluit A. (2018). Antibacterial and immunogenic behavior of silver coatings on additively manufactured porous titanium. Acta Biomater..

[45] DeMuth D.R., Irvine D.C., Costerton J.W., Cook G.S., Lamont R.J. (2001). Discrete Protein Determinant Directs the Species-Specific Adherence of Porphyromonas gingivalis to Oral Streptococci. Infect. Immun..

[46] Maeda K., Nagata H., Ojima M., Amano A. (2015). Proteomic and Transcriptional Analysis of Interaction between Oral Microbiota Porphyromonas gingivalis and Streptococcus oralis. J. Proteome Res..

[47] Paju S., Pussinen P.J., Suominen-Taipale L., Hyvönen M., Knuuttila M., Könönen E. (2009). Detection of Multiple Pathogenic Species in Saliva Is Associated with Periodontal Infection in Adults. J. Clin. Microbiol..

[48] Daghighi S., Sjollema J., Jaspers V., De Boer L., Zaat S.A.J., Dijkstra R.J.B., Van Dam G.M., Van Der Mei H.C., Busscher H.J. (2012). Persistence of a bioluminescent Staphylococcus aureus strain on and around degradable and non-degradable surgical meshes in a murine model. Acta Biomater..

[49] Rais B., Köster M., Rahim M.I., Pils M., Seitz J., Hauser H., Wirth D., Mueller P.P. (2016). Evaluation of the inflammatory potential of implant materials in a mouse model by bioluminescent imaging of intravenously injected bone marrow cells. J. Biomed. Mater. Res. Part A.

[50] Kesavalu L., Holt S.C., Ebersole J.L. (1998). Virulence of a polymicrobic complex, Treponema denticola and Porphyromonas gingivalis, in a murine model. Oral Microbiol. Immunol..

[51] Polak D., Shapira L., Weiss E.I., Houri-Haddad Y. (2012). The role of coaggregation between Porphyromonas gingivalis and Fusobacterium nucleatum on the host response to mixed infection. J. Clin. Periodontol..

[52] Feuille F., Ebersole J.L., Kesavalu L., Stepfen M.J., Holt S.C. (1996). Mixed infection with Porphyromonas gingivalis and Fusobacterium nucleatum in a murine lesion model: Potential synergistic effects on virulence. Infect. Immun..

[53] Ebersole J.L., Feuille F., Kesavalu L., Holt S.C. (1997). Host modulation of tissue destruction caused by periodontopathogens: Effects on a mixed microbial infection composed of Porphyromonas gingivalisand Fusobacterium nucleatum. Microb. Pathog..

[54] Polak D., Wilensky A., Shapira L., Halabi A., Goldstein D., Weiss E.I., Houri-Haddad Y. (2009). Mouse model of experimental periodontitis induced by Porphyromonas gingivalis/Fusobacterium nucleatum infection: Bone loss and host response. J. Clin. Periodontol..

[55] Kesavalu L., Sathishkumar S., Bakthavatchalu V., Matthews C., Dawson D., Steffen M., Ebersole J.L. (2007). Rat Model of Polymicrobial Infection, Immunity, and Alveolar Bone Resorption in Periodontal Disease. Infect. Immun..

