# Supplementary material for: Non-Invasive Luciferase Imaging of Type I Interferon Induction in a Transgenic Mouse Model of Biomaterial Associated Bacterial Infections: Microbial Specificity and Inter-Bacterial Species Interactions
Source: Microorganisms. 2020 Oct 21;8(10):1624. doi: 10.3390/microorganisms8101624 (PMC7589032; doi:10.3390/microorganisms8101624)
Supplement: Supplementary file 1 [file microorganisms-08-01624-s001.pdf]

SUPPLEMENTARY INFORMATION

Non-invasive luciferase imaging of type I interferon induction in a transgenic mouse model of biomaterials-associated bacterial infections: microbial specificity and inter-bacterial interactions

Muhammad Imran Rahim[1,5*], Andreas Winkel[1,5], Stefan Lienenklaus[2], Nico S. Stumpp[1,5], Szymon P. Szafrański [1,5], Nadine Kommerein[1,5], Elmar Willbold[3,5], Janin Reifenrath[3,5], Peter P. Mueller[4], Michael Eisenburger[1], Meike Stiesch[1]

[1] Department of Prosthetic Dentistry and Biomedical Materials Science, Hannover Medical School, Carl-Neuberg-Strasse 1, 30625 Hannover, Germany; Winkel.Andreas@mh-hannover.de (A.W.); Stumpp. Nico@mh-hannover.de (N.S.S.); Szafranski.Szymon@mh-hannover.de (S.P.S.); Kommerein.Nadine@mh-hannover.de (N.K.); Eisenburger.Michael@mh-hannover.de (M.E.); Stiesch.Meike@mh-hannover.de (M.S.)

[2] Institute of Laboratory Animal Science, Hannover Medical School, Carl-Neuberg-Strasse 1, 30625 Hannover, Germany; Lienenklaus.Stefan@mh-hannover.de (S.L.)

[3] Department of Orthopedic Surgery, Hannover Medical School, Carl-Neuberg-Strasse 1, 30625 Hannover, Germany; Willbold.Elmar@mh-hannover.de (E.W.); Reifenrath.Janin@mh-hannover.de (J.R.)

[4] Department of Chemical Biology, Helmholtz Center for Infection Research, Inhoffenstrasse7, 38124 Braunschweig, Germany; Peter-Paul.Mueller@helmholtz-hzi.de (P.P.M.)

[5] Lower Saxony Centre for Biomedical Engineering, Implant Research and Development (NIFE), Stadtfelddamm 34, 30625 Hannover, Germany

Corresponding Author

*Dr. Muhammad Imran Rahim

Department of Prosthetic Dentistry and Biomedical Materials Science

Hannover Medical School, Hannover, Germany

Email: Rahim.Muhammad@mh-hannover.de

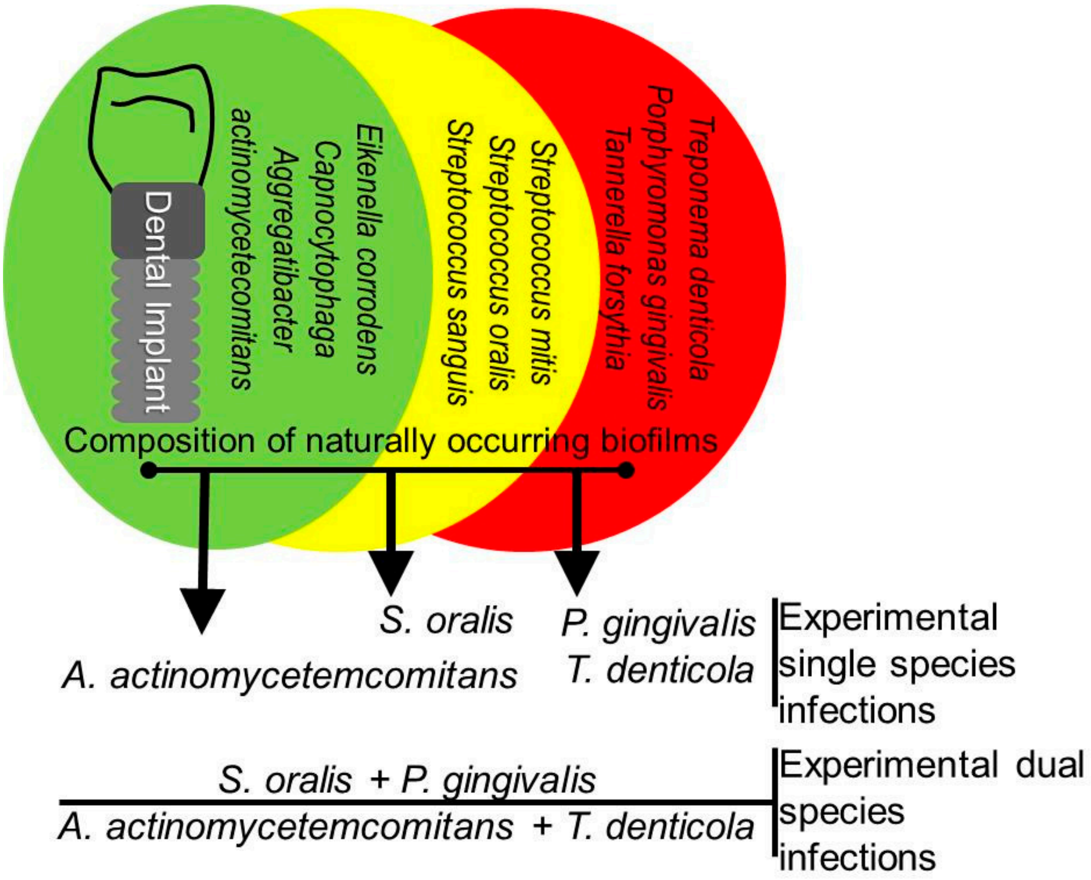

Figure S1. Selection of periodontal pathogens to investigate inflammatory responses against implant-related infections. According to Socransky's classification of periodontal pathogens (different colors), *Streptococcus oralis* (So) and *Aggregatibacter actinomycetemcomitans* (*Aa*) are associated with acute periodontal diseases (yellow and green). *Treponema denticola* (*Td*) and *Porphyromonas gingivalis* (*Pg*) are detected in chronic periodontal diseases. Single species infections were done with the indicated bacteria representative of each group. Dual species infections were done by mixing *S. oralis* or *A. actinomycetemcomitans* from acute group with *T. denticola* or *P. gingivalis* from the chronic group.

Supplementary Table 1 Classification of animal groups employed for the current study

Animal Groups		Procedure	Group size	Implants per animal	Total implants analyzed
1	Sterile implant	implantation in subcutaneous tissue	3	3	9
2	implant + *S. oralis*	Implantation of titanium and then infection with *S. oralis*	3	3	9
3	*S. oralis* infection	*S. oralis* infection in sham surgical pouches in subcutaneous tissues	3	-	-
4	Implant + *A. actinomycetemcomitans*	Implantation of titanium and then infection with *A. actinomycetmcomitans*	3	3	9
5	*A. actinomycetemcomitans* infection	*A. actinomycetmcomitans* infection in sham surgical pouches in subcutaneous tissues	3	-	-
6	Implant + *P. gingivalis*	Implantation of titanium and then infection with *P. gingivalis*	3	3	9
7	*P. gingivalis* infection	*P. gingivalis* infection in sham surgical pouches in subcutaneous tissues	3	-	-
8	Implant + *T. denticola*	Implantation of titanium and then infection with *T. denticola*	3	3	9
9	*T. denticola* infection	*T. denticola* infection in sham surgical pouches in subcutaneous tissues	3	-	-
10	Implant + *T. denticola-A. actinomycetmcomitans*	Implantation of titanium and then infection with mixture of *T. denticola-A. actinomycetmcomitans*	3	3	9
11	*T. denticola-A. actinomycetemcomitans* infection	*T. denticola-A. actinomycetmcomitans* infection in sham surgical pouches in subcutaneous tissues	3	-	-
12	Implant + *S. oralis-P.gingivalis*	Implantation of titanium and then infection with mixture of *S. oralis-P.gingivalis*	3	3	9
13	*S. oralis-P.gingivalis* infection	*S. oralis-P.gingivalis* infection in sham surgical pouches in subcutaneous tissues	3	-	-

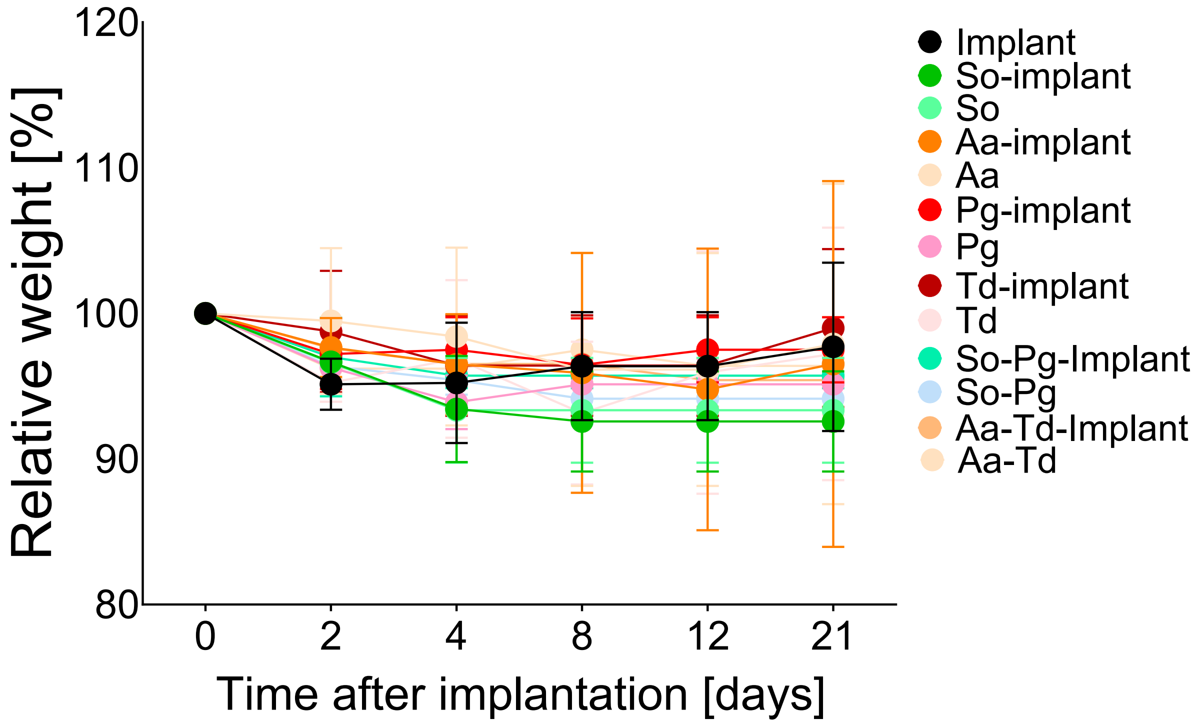

Figure S2. Relative body weight of mice. The body weight of mice immediately after performing surgical implantations was arbitrarily set to 100% and repeated determined at the indicated time points. Abbreviations: implant, mice carrying uninfected implants; So-implant, mice carrying implants infected with *S. oralis*; So, mice operated to create subcutaneous pouches and then infected with *S. oralis*; Aa-implant, mice carrying implants infected with *A. actinomycetemcomitans*; Aa, mice operated for subcutaneous pouches and then infected with *A. actinomycetemcomitans*; Pg-implant, mice carrying implants infected with *P. gingivalis*; Pg, mice operated for subcutaneous pouches that were infected with *P. gingivalis*; Td-implant, mice carrying implants infected with *T. denticola*; Td, mice operated for subcutaneous pouches and then infected with *T. denticola*; So-Pg-implant, mice carrying implants infected with *S. oralis* and *P. gingivalis*; So-Pg, mice operated for subcutaneous pouches and then infected with *S. oralis* and *P. gingivalis*; ; Aa-Td-implant, mice carrying implants infected with *A. actinomycetemcomitans* and *T. denticola*; Aa-Td, mice operated for subcutaneous pouches and then infected with *A. actinomycetemcomitans* and *T. denticola*.

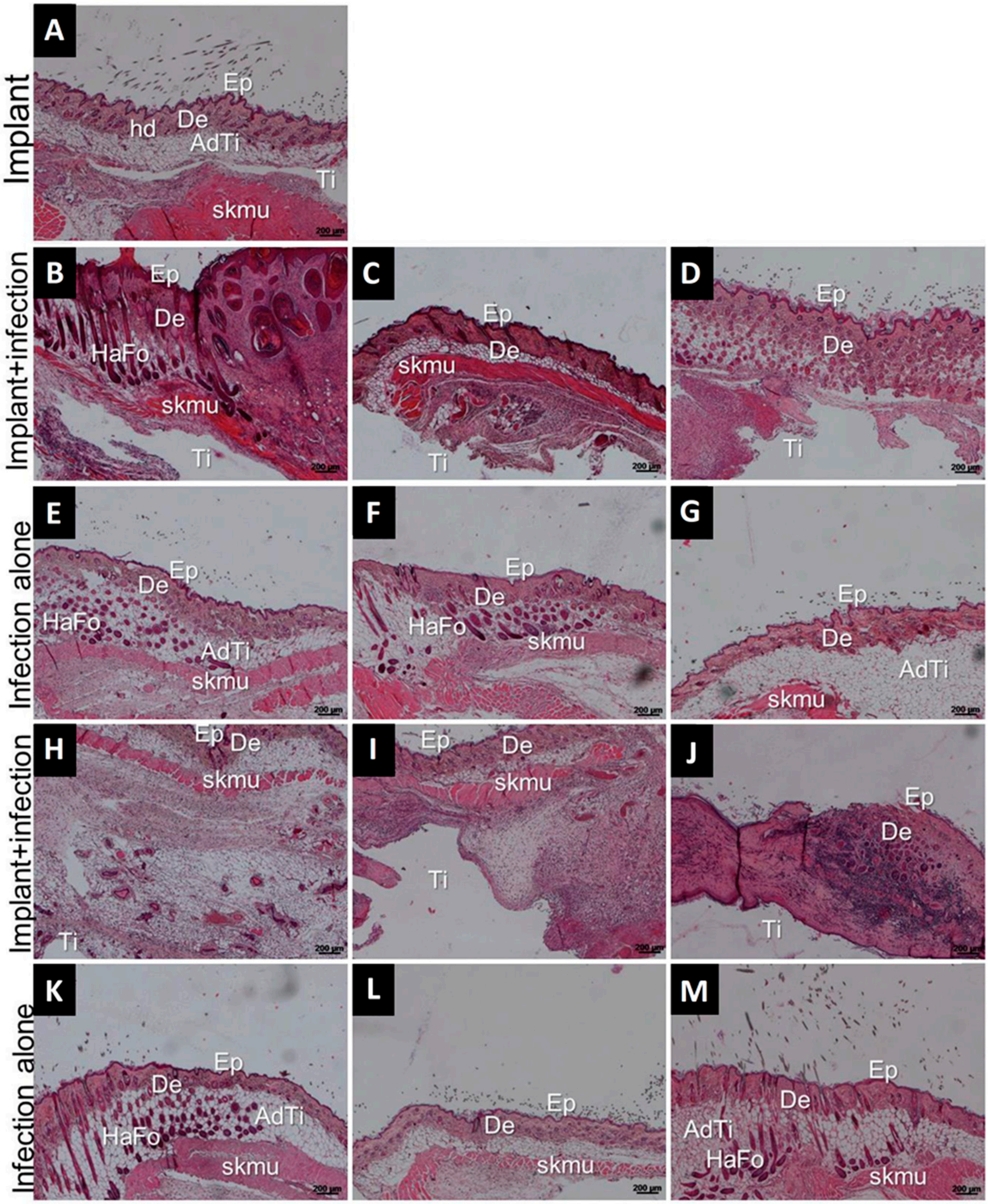

Figure S3. Histological evaluation of skin around sterile implants, infected implants and infected tissue after 21 days. Few immune cells are visible around the areas (Ti) marking sterile implants

which were removed before the histological staining (A). Large numbers of inflammatory cells (blue nuclei) were present at tissues-implant (Ti) interfaces infected with *S. oralis* (B), *A. actinomycetemcomitans* (C), *P. gingivalis* (D) or *T. denticola* (H). Tissue infections without implants showed no signs of inflammation (E, F, G, and K). Tissue-implant interfaces after infections with dual species *S. oralis-P. gingivalis* (I) and *A. actinomycetemcomitans-T.denticola* (J) showed huge recruitment of inflammatory cells (blue nuclei). Dual species infected tissues without implants did not exhibit high recruitment of inflammatory cells (L and M). Abbreviations: Ep, epidermis; De, dermis, hd, hypodermis; skmu, skeletal muscle; HaFo, Hair follicle; HaBu, Hair bubble; AdTi; Adipose tissue; Ti, site of titanium implant.
